# Expression of Rab1A is upregulated in human lung cancer and associated with tumor size and T stage

**DOI:** 10.18632/aging.101087

**Published:** 2016-11-29

**Authors:** Xinxin Wang, Feng Liu, Xiaoyu Qin, Tinglei Huang, Bo Huang, Yanjie Zhang, Bin Jiang

**Affiliations:** ^1^ Oncology Department, Shanghai Ninth People's Hospital, Shanghai Jiaotong University School of Medicine, Shanghai 201999, China

**Keywords:** lung carcinoma, Rab1A, immunohistochemistry, mTOR, cell signaling pathways

## Abstract

Rab1A expression is associated with malignant phenotypes in several human tumors; however, the role of Rab1A in lung cancer is still unclear. In this study, we attempted to establish the role of Rab1A in major human lung cancer subtypes. Rab1A expression in different histological types of human lung cancer was analyzed in lung cancer tissues with paired adjacent noncancerous tissues and a large panel of lung cancer cell lines. The effect of Rab1A expression on multiple cancer-associated signaling pathways was also examined. The results demonstrated that Rab1A was significantly overexpressed in the different histological types of lung cancer as compared to non-cancerous tissues, and Rab1A expression was correlated with tumor volume and stage. In a large panel of lung cancer cell lines, high Rab1A expression was observed as compared to a normal lung/bronchus epithelial cell line. However, Rab1A protein levels were not correlated with mTORC1 (P-S6K1), mTORC2 (P-AKT), MEK (P-ERK), JNK (P-c-Jun) or p38MAPK (P-MK2) signaling. Rab1A knockdown had no effect on mTOR signaling or cell growth. These data suggested that Rab1A may be involved in the pathogenesis of human lung cancer in an mTOR- and MAPK-independent manner.

## INTRODUCTION

Lung cancer is the leading cause of cancer-related death worldwide, with 1.8 million new cases and 1.6 million deaths reported in 2012 [1-3]. Only 16% of early-stage lung cancer is diagnosed, and the overall 5-year survival rate is only 15% [4]. Even after curative resection and systemic chemotherapy, a significant number of lung cancer patients die due to recurrence and distant metastases. Therefore, the identification of novel therapeutic targets to improve survival of lung cancer patients is urgently needed.

Rab1A, a small GTPase, has been well established to mediate vesicular trafficking between the endoplasmic reticulum (ER) and Golgi apparatus [5]. It is a highly conserved protein, which has been identified in 158 different organisms, ranging from yeast to humans [6, 7]. Recently, a growing body of evidence has suggested that the Rab1A protein has additional functions beyond its role in vesicular transport, including nutrient sensing [8] and signaling [9], cell migration [10] and regulation of autophagy [11]. Moreover, aberrant expression of Rab1A has been linked to a range of human diseases including cardiomyopathy [12], Parkinson's disease [13] and cancer [8, 14-19].

Rab1A has been identified as a colorectal oncogene, and elevated expression of Rab1A has been reported in multiple cancer types, including colorectal cancer (CRC) [8], hepatocellular carcinoma (HCC) [18], glioma [14], prostate cancer [15, 17], tongue squamous carcinoma [16] and cervical cancer [19]. Overexpression of Rab1A is correlated with a poor prognosis and has been proposed to promote tumor progression by activating the mTORC1 signaling pathway in CRC and HCC, indicating that Rab1A might be a valuable therapeutic target for personalized therapy [8]. Despite the importance of Rab1A in human malignancies, to date, Rab1A has not been studied in the context of lung cancer. In this study, we investigated Rab1A expression in tissues and cell lines from different lung cancer subtypes, and assessed the relationships between Rab1A expression and clinical parameters as well as key cancer cell signaling pathways.

## RESULTS

### Rab1A is highly expressed in lung cancer tissues

**Figure 1:**
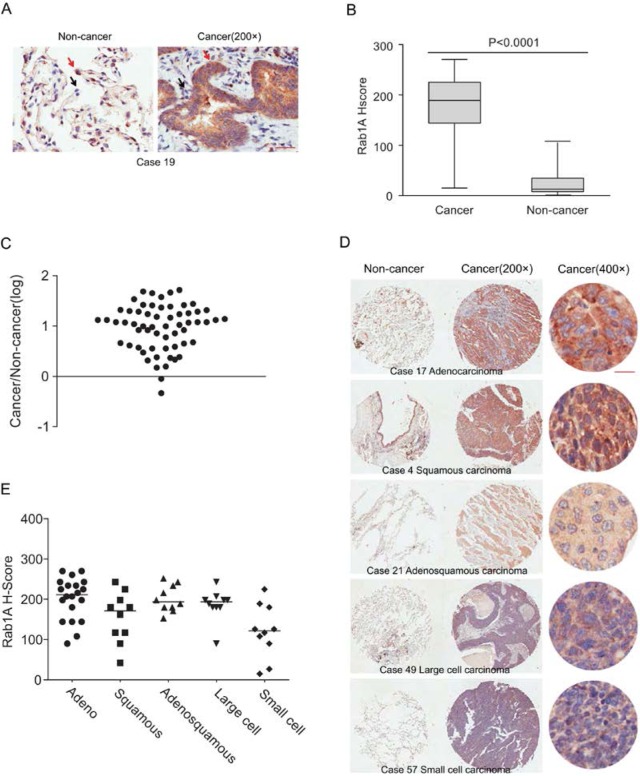
Rab1A is frequently overexpressed in different histological types of lung cancer (**A**) IHC staining of human lung cancer tissue and noncancerous tissues. Shown are representative images of stained tumor and non-cancerous tissue sections. Red arrowhead: high Rab1A staining; black arrowhead: low Rab1A staining. Scale bar = 50 μm. (**B**) Box plot graph showing the statistical analysis of Rab1A expression in lung cancer and paired non-cancerous tissues. (**C**) Scatter plot showing Rab1A staining levels in individual tumors as a ratio of Rab1A staining in lung cancer to the paired non-cancerous tissue. (**D**) IHC staining of human lung cancer tissue microarray and paired noncancerous tissues. Shown are stained tumor and non-cancerous tissue sections representative of different histological types. Scale bar = 20 μm. (**E**) Scatter plot showing levels of Rab1A staining in different histological types of lung cancer as a ratio of Rab1A staining in cancer tissues to paired non-cancerous tissue.

Immunohistochemical staining was performed to evaluate Rab1A expression in 60 lung cancer tissues and paired adjacent noncancerous tissues. Positive staining appeared brown. Rab1A was predominantly expressed in the cytoplasm, and the percentage of nuclear expression is very low (less than 5%) in both noncancerous tissues and cancerous tissues. In contrast to the noncancerous tissues, Rab1A was significantly highly expressed in tumor cells (Figure 1A), while faint staining was present in stromal and lymphoid cells in paired noncancerous tissues. When using the median H score of 67.5 as a cutoff value, the overall rate of positive staining in lung cancer tissues (95%) was significantly higher than in paired noncancerous tissues (5%; P = 0.000; Table 1). Compared with matched noncancerous tissues (median H score = 15), Rab1A staining was much stronger in lung cancer tissues (median H score = 189; Figure 1B). On an individual basis, Rab1A scored higher in approximately 96.67% (58/60) of tumors than in the corresponding noncancerous tissues, although Rab1A staining was remarkably variable, differing by as much as 100-fold between different tumor samples (Figure 1C). For different histological subtypes of lung cancer, Rab1A showed similar expression patterns (Figure 1D) with a 100% positive staining rate in adenocarcinoma, adenosquamous carcinoma and large cell lung cancer tissues (Table 2). The H score in the adenocarcinoma group (median, 211.5) was higher than others histological types while the small cell lung cancer cohort showed the lowest H score (median, 121.5; Figure 1E).

**Table 1:** Rab1A expression in lung cancer and paired noncancerous tissues

Tissue type	Cases	Rab1A expression		P value
		Positive N (%)	Negative N (%)	
Lung cancer	60	57 (95)	3 (5)	0.000
Paired noncancerous	60	3 (5)	57 (95)	

P = 0.000; cancer vs. paired noncancerous determined by Fisher's exact test

**Table 2:** Rab1A expression in five pathological subtypes of lung cancer

Pathological subtype	Cases	Rab1A expression		P value
		Positive N (%)	Negative N (%)	
Adenocarcinoma	20	20 (100)	0 (0)	
Squamous carcinoma	10	9 (90)	1 (10)	
Adenosquamous carcinoma	10	10 (100)	0 (0)	0.000
Large cell lung cancer	10	10 (100)	0 (0)	
Small cell lung cancer	10	8 (80)	2 (20)	

All P values = 0.000; cancer vs. paired noncancerous as determined by Fisher's exact test.

### Rab1A expression is correlated with clinical parameters

To further delineate the clinical significance of Rab1A expression, we examined the relationship between Rab1A staining and widely recognized clinicopathologic factors. Cases were divided into high and low Rab1A staining groups based on the median H score of 180 in cancer tissues. The Kruskal-Wallis test showed that Rab1A staining is significantly correlated with tumor size (P = 0.002), and T stage (P = 0.039), as shown in Table 3. Large tumors and late T stage tumors consistently displayed higher Rab1A expression when compared with small tumor size and early T stage, indicating that Rab1A may promote tumor growth and progression. However, there were no significant associations observed between Rab1A expression and age, gender, lymph node status and AJCC stage.

**Table 3:** Association between Rab1A expression and clinicopathologic features

Clinicopathologic feature	Cases	Rab1A expression		P value
		High	Low	
Pathological grade				0.636
I	14	6	8	
II	29	14	15	
III	17	8	9	
Gender				0.101
Male	45	22	23	
Female	15	6	9	
Age (years, Median = 62)				0.395
≤62	31	12	19	
>62	29	16	13	
Tumor size (cm^3^, Median = 24)				0.002
≤24	31	9	22	
>24	29	19	10	
T stage				0.039
T1-T2	45	20	25	
T3-T4	15	8	7	
Lymph node status				0.749
N0	28	13	15	
N1-N2	32	18	14	
AJCC stage				0.779
I- II	34	17	17	
III-IV	26	14	12	

Cases were divided by high Rab1A staining (H score ≥ 180) and low Rab1A staining (H score < 180). The Kruskal-Wallis test was performed and P values are shown.

### Rab1A is highly expressed in lung cancer cell lines but not correlated with expression of key signaling pathways

**Figure 2:**
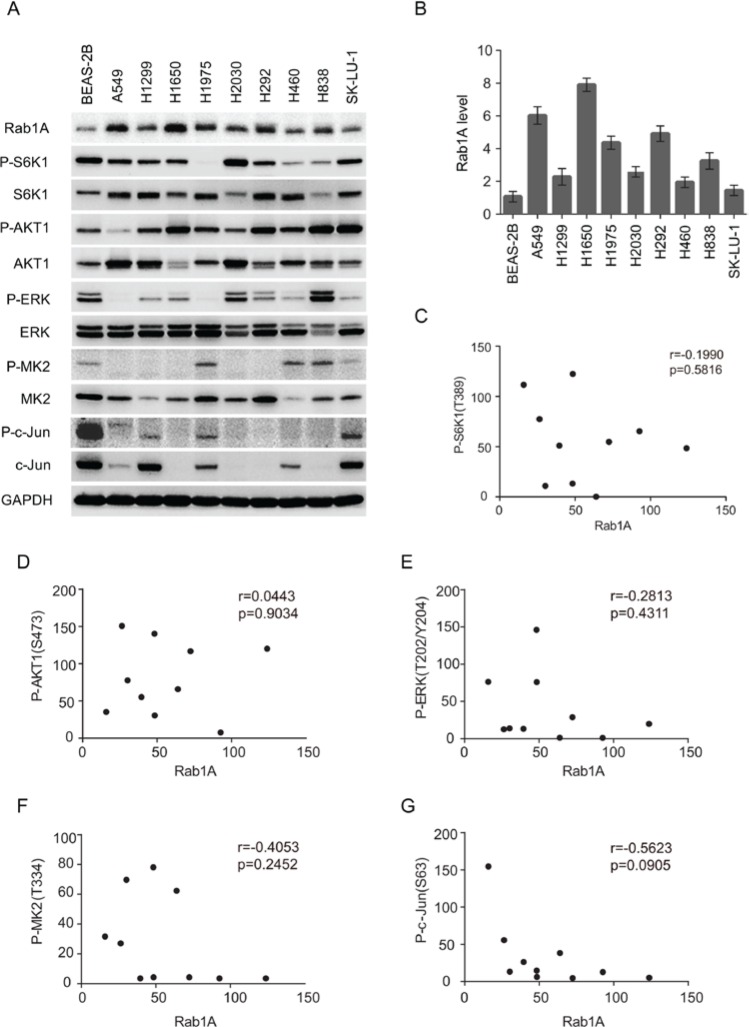
Rab1A is overexpressed in lung cancer cell lines but is not correlated with mTORC or MAPK expression (**A**) Expression of Rab1A, P-S6K, S6K, P-AKT, AKT, P-ERK, ERK, P-MK2, MK2, P-c-Jun and c-Jun was examined in a panel of lung cancer cell lines by immunoblot. GAPDH served as a loading control. (**B**) Quantitative analysis of Rab1A protein levels in cell lines. The densitometry results for each Rab1A band were normalized to GAPDH, and the ratios between cancer cell lines and BEAS-2B were calculated and results represented in a histogram. Data represent means ± SD of three independent experiments. (**C-F**) Correlation plots of the expression level of Rab1A and P-S6K1 (T389; **C**), P-AKT (S473; **D**), P-ERK (T202/Y204; **E**), P-MK2 (T334; **F**) and P-c-Jun (S63; **G**). Shown are arbitrary units.

We further examined Rab1A expression in a panel of lung cancer cell lines. Compared with the immortalized lung/bronchus epithelial cell line BEAS-2B, Rab1A is highly expressed in all lung cancer cell lines with great variability, ranging from a 6-fold increase in the A549 and H1650 cell lines to a 1.5-fold increase in H460 and SK-LU-1 cells lines (Figure 2A and B). These findings are consistent with Rab1A expression levels in lung cancer tissues. Because Rab1A has been reported to be correlated with hyperactive mTORC1 signaling in human colorectal cancer [8], we examined whether Rab1A expression was correlated with mTORC1 signaling in lung cancer. However, no significant correlation between Rab1A and P-S6K1 (T389) was observed in lung cancer cell line panels. We further examined substrates of other key cancer cell signaling pathways, including P-AKT (S473), P-ERK (T202/Y204), P-C-JUN (S63) and P-MK2 (T334), but no correlation between expression of these proteins and Rab1A expression was observed (Figure 2C-G), indicating that, under physiological conditions, Rab1A expression is not correlated with the basal expression levels of the mTORC1, mTORC2, MEK, JNK and p38MAPK signaling pathways in lung cancer.

### Rab1A knockdown has no effect on mTOR signaling or lung cancer cell growth

**Figure 3:**
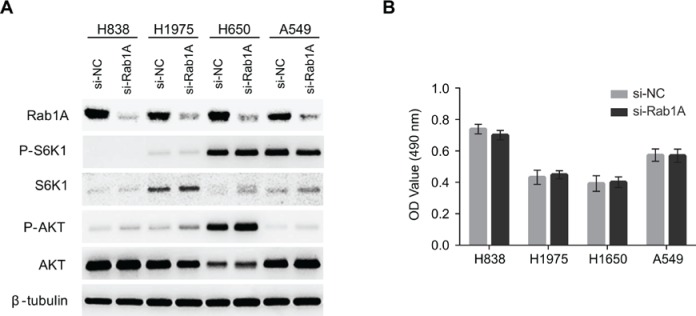
Rab1A does not regulate mTOR signaling or cell growth in lung cancer (**A**) The effects of Rab1A knockdown was examined in H838, H1975, H650 and A549 cells by immunoblot. Levels of P-S6K1 (T389), S6K, P-AKT (S473) and AKT are shown. β-tubulin was used as a loading control. (**B**) Rab1A was knocked down in H838, H1975, H650 and A549 cells. The relative growth of these cells was analyzed using an MTT assay. Data represent means ± SD of three independent triplicate experiments. si-NC, negative control siRNA; si-Rab1A, Rab1A-specific siRNA.

It has been reported that Rab1A knockdown dramatically attenuated mTORC1 signaling and cell growth in CRC and HCC. Therefore, we examined whether Rab1A knockdown had similar effects in human lung cancers. Although Rab1A was successfully knocked down in a panel of lung cancer cell lines, no changes were observed with regards to mTORC1 (P-S6K1(T389)) or mTORC2 (P-AKT (S473)) signaling (Figure 3A). Consistent with these findings, cell growth was not affected by Rab1A knockdown in an MTT assay (Figure 3B).

## DISCUSSION

Elevated levels of Rab1A DNA, mRNA or protein have been reported previously in human CRC [8], gliomas [14], HCC [18], prostate cancer [15] and tongue squamous carcinomas [16]. Our study for the first time shows that Rab1A is significantly overexpressed in lung cancer. These results indicate that Rab1A overexpression is widespread in human malignancies, consistent with its oncogenic function. In addition, in lung cancer, Rab1A expression levels were not correlated with histological type; different lung cancer subtype tissues and cell lines all showed significant Rab1A overexpression, suggesting that aberrant Rab1A expression is universal in lung cancers. It has been reported that Rab1A overexpression is correlated with disease progression and prognosis in CRC and HCC [8,18]. Here we report that Rab1A overexpression is correlated with tumor volume and stage in lung cancer. This result indicates that Rab1A may contribute to lung cancer progression, and further emphasizes the important role of Rab1A in human malignancies.

Recent studies have shown that Rab1 is a regulator of several central signal transduction pathways, particularly the mTOR pathway [6,21]. Rab1A overexpression is a driver of mTORC1 signaling and mTORC1-dependent growth in CRC by regulating the interaction of mTORC1 with the small GTPase Rheb, which activates mTORC1 activity on the Golgi surface. However, unlike in CRC, Rab1A overexpression is not correlated with basal mTORC1 (P-S6K1) or mTORC2 (P-AKT (S473)) signaling in lung cancer cells, and Rab1A knockdown did not significantly alter mTORC1 or mTORC2 activity. In addition, Rab1A knockdown had no effect on cell growth or the induction of apoptosis (data not shown), which is consistent with a lack of effect on mTORC1 and mTORC2 signaling. These results indicate that, although Rab1A overexpression is a general phenomenon in human malignancies, its function and mechanism of action are highly variable in different cancer types. Rab1A did not regulate mTOR signaling in human lung cancer. It would be of interest to investigate whether Rab1B, a paralog of Rab1A, could play a major role in mTOR regulation in lung cancer.

We also analyzed a panel of lung cancer cell lines for additional key signaling pathways including mitogen-activated protein kinase (MAPK) pathways, which are often deregulated in human cancers [22-24]. Three distinct MAPK cascades, MEK/ERK, JNK/c-Jun and p38/MK2 were investigated, but no significant correlation with Rab1A was observed, indicating that MAPK pathways may not be involved in the mechanisms by which Rab1A contributes to lung cancer.

In light of the present data considered in the context of previous findings, we can hypothesize that Rab1A overexpression is associated with clinical pathological parameters of human lung cancer. Because this study examined limit number of cell lines from different histological subtypes, to achieve a sound conclusion, correlation between major cancer signaling pathways and the malignant role of Rab1A needs to be further evaluated in a larger sample set of cancer tissues/cancer cell lines from specific histological subtype of lung cancer. The mechanisms by which Rab1A overexpression contributes to lung cancer, including cell cycle progressing and promoting migration and metastasis, are under investigation.

## MATERIALS AND METHODS

### Tissue array and immunohistochemistry

Sixty de-identified lung cancer tissues with paired adjacent noncancerous tissues were collected from the hospital between January 2007 and March 2008 and developed into tissue arrays by Shanghai OUTDO Biotech (Shanghai, China). Twenty adenocarcinomas, 10 squamous carcinomas, 10 adenosquamous carcinomas, 10 large cell carcinomas and 10 small cell carcinomas were included. The study protocols were approved by the SJTUSM (Shanghai Jiao Tong University School of Medicine) Ethics Committee. All procedures adhere to the BRISQ Guidelines for reporting research on human biospecimens.

Immunohistochemical detection of Rab1A was performed using a streptavidin-biotin complex method as described previously [8]. The primary antibody against Rab1A (Proteintech Group) was used at a concentration of 1:100. To score a tumor cell as positive, cytoplasmic or nuclear staining was performed. For quantitative analysis, a Histo (H) score was calculated based on the staining intensity and percentage of stained cells using Aperio ScanScope systems (Vista, CA, USA). The intensity score was defined as follows: 0, no appreciable staining in cells; 1, weak staining in cells comparable with stromal cells; 2, intermediate staining; 3, strong staining. The fraction of positive cells was scored as 0%-100%. The H score was calculated as previously described, by multiplying the intensity score and the fraction score [8], producing a total range of 0-300. Based on the distribution of the data, a cutoff of 67.5 was used for Rab1A positivity. Tissue sections were examined and scored separately by two independent investigators blinded to the clinicopathologic data.

### Cell lines and culture conditions

Immortalized bronchial epithelial cells (BEAS-2B) and nine human lung cancer cell lines (A549, H1299, H1650, H1975, H2030, H292, H460, H838, SK-LU-1) were purchased from ATCC (American type culture collection). All cell lines were confirmed to be free of mycoplasma contamination. BEAS-2B and H292 cells were cultured in Dulbecco's Modified Eagle Medium with 10% fetal bovine serum (FBS). A549, H1299, H1650, H1975, H2030, H460, H838 and SK-LU-1 cells were cultured in RPMI 1640 medium with 10% FBS. All cell lines were maintained at 37°С in a 5% CO_2_ incubator. Cell lines were immediately expanded upon receipt and aliquots frozen to allow the cell lines to be restarted every three to four months from the same batch of cells. Cell phenotypes were verified in every experiment.

### siRNA transfection

Small interfering RNAs (siRNAs) targeting human Rab1A was synthesized by GenePharma (Shanghai, China) and have been validated in our previous studies [8]. Sequences: Rab1A siRNA, AAU AAC UGG AGG UGA UUG UUC, Negative control siRNA: UUC UCC GAA CGU GUC ACG UTT. Transfections were performed on a panel of lung cancer cell lines. Cells were incubated with siRNA oligonucleotides and Lipofectamine 3000 (ThermoFisher, Pittsburgh, PA) transfection reagent for 12 h. The cell culture medium was then replenished with fresh culture medium and the cells were analyzed.

### MTT assay

To determine the effects of Rab1A knockdown, cell growth was analyzed using an MTT assay as previously described [20]. Cells were harvested 12 h post-transfection and re-seeded in 96-well plates at an initial density of 3 × 10^3^ cells/well. Assays were performed at 48 h post-seeding according to the manufacturer's protocols. Data represent the mean ± SD (absorbance value at 490 nm) from three independent triplicate experiments.

### Antibodies and immunoblotting

Cells were harvested 48 h post-transfection and subjected to immunoblotting as described previously [8]. Reagents were obtained from the following sources: antibody for Rab1A, Proteintech Group (Rosemont, IL); antibodies for P-S6K1(T398), S6K1, P-AKT(S473), AKT, P-ERK(T202/Y204), ERK, P-MK2(T334), MK2 and Tubulin, Cell Signaling Technology (Danvers, MA). HRP-labeled GAPDH, anti-mouse and anti-rabbit antibodies were purchased from Santa-Cruz Biotechnology (Dallas, Texas). The EDTA-free Complete Protease Inhibitor Cocktail and PhosSTOP were obtained from Roche (Basel, Switzerland). All primary antibodies were confirmed to be reactive only to the targets by the manufacturer and used at 1:1000, and secondary antibodies were used at 1:10000. The data represent at least three independent experiments.

### Statistical analysis

Statistical analyses were carried out using SPSS 20.0 software. The statistical analysis of numeration data was performed using Pearson's chi-square test or Fisher's exact chi-square test. The comparisons of continuous data between groups were performed using the Kruskal-Wallis test. A nonparametric Spearman's correlation test was performed to analyze the correlation between Rab1A and P-S6K1, P-AKT1, P-ERK, P-MK2 and P-c-Jun expression levels. All statistical tests were conducted at a two-sided significance level of 0.05.
